# Reduction in Operating Room Airborne Particle Burden and Time-Dependent Contamination of Sterile Instrument Trays With the Use of a Novel Air Filtration System

**DOI:** 10.7759/cureus.26864

**Published:** 2022-07-14

**Authors:** Fady Y Hijji, Andrew D Schneider, Jeffrey T Reeves, Michael L Wilson, Logan Nye, Joseph G Lyons, Michael J Prayson, Louis J Rubino

**Affiliations:** 1 Orthopaedic Surgery, Boonshoft School of Medicine, Dayton, USA

**Keywords:** orthopedic infections, airborne infection control, microbial contamination, air-contamination, operating room safety, peri-prosthetic joint infection, prosthetic infection, post surgical infection, airborne contamination, ultraviolet filtration

## Abstract

Introduction

Postoperative infections represent a substantial burden to patients and healthcare systems. To improve patient care and reduce healthcare expenditures, interventions to reduce surgical infections must be employed. The crystalline C-band ultraviolet (UV-C) air filtration technology (Aerobiotix Inc., Miamisburg, OH, USA) has been designed to reduce airborne bioburden through high-quality filtration and germicidal irradiation. The purpose of this study was to assess the ability of a novel UV-C air filtration device to reduce airborne particle counts and contamination of surgical instrument trays in an operating room (OR) setting.

Materials and methods

Thirty sterile instrument trays were opened in a positive-air-flow OR. The trays were randomly assigned to one of two groups (UV-C or control, n=15 per group). In the UV-C group, the UV-C filtration device was used and in the control, it was not. All trays were opened with the use of a sterile technique and left exposed in the OR for four hours. Air was sampled by a particle counter to measure the numbers of 5µm and 10µm particles. Culture specimens were obtained from the trays to assess for bacterial contamination. Outcome data were collected at 30-minute intervals for the duration of the four-hour study period.

Results

Use of the UV-C device resulted in statistically significant reductions in the numbers of 5µm (average of 64.9% reduction when compared with the control, p<0.001) and 10µm (average of 65.7% reduction when compared with the control, p<0.001)-sized particles detectable in the OR. There was no significant difference in the overall rates of contamination (33.3% in the control group vs. 26.7% in the UV-C group, p=1.0) or the time to contamination (mean survival of 114 minutes in the control group vs. 105 minutes in the UV-C group, p=0.72) of surgical instrument trays with the use of the UV-C device.

Conclusions

The results demonstrate that the UV-C filtration device can successfully reduce airborne bioburden in standard ORs, suggesting that it may have the potential to reduce the risk for wound and hardware infections. Further clinical trials are necessary to better determine the effect of this air filtration system on postoperative infection rates.

## Introduction

Postoperative infection is one of the most challenging complications following orthopedic surgery today. Implant-associated and prosthetic infections represent a substantial burden to patients and healthcare systems [1,2]. The health care costs of prosthetic joint infections alone are enormous, in the billions of dollars annually in the United States alone [2,3]. Implant-associated infections are also associated with substantial morbidity and mortality, with five-year mortality rates after the diagnosis reported at greater than 25% [4,5]. In order to improve patient outcomes and reduce healthcare expenditures, interventions to reduce surgical infections must be employed.

Airborne contamination has been attributed as a potential cause of surgical site infections for decades [6-9]. Aerosolized particles including dust, skin scales, and respiratory particulates arising from patients and surgical staff frequently carry a variety of organisms including *Staphylococcus aureus* and Coagulase-negative staphylococci [7]. These particles eventually settle and can contaminate surgical instruments and/or surgical wounds. Increased airborne particle counts in the operating room (OR) have previously been associated with implant contamination and surgical site infections [1,9-11]. In an effort to minimize intraoperative airborne contamination, hospitals have employed a variety of technologies including high-efficiency particulate arrestance (HEPA) filtration, positive air pressurization, and surgical helmet systems [1]. Guidelines have also been developed to improve OR air quality, however, many of these guidelines do not provide specific criteria to adequately eliminate microbial aerosols or minimum particle count standards [1,12]. Additionally, necessary OR activity such as personnel traffic and surgical gowning have been demonstrated to increase viable airborne particulates that are not well controlled by current OR air systems [13-16]. As a result, poor OR air quality has remained a healthcare issue and a contributing factor in surgical infections.

In order to mitigate airborne contamination and surgical infections, multiple innovative technologies are emerging. One such technology is the crystalline C-band UV (UV-C) air filtration (Aerobiotix Inc., Miamisburg, OH, USA), designed to reduce contamination through high-quality filtration and germicidal irradiation [17]. In this context, the purpose of the present study was to evaluate the ability of a UV-C air filtration device to reduce airborne particles and bacteria in an OR setting. Specifically, this study sought to assess the effects of a specific UV-C filtration device on particle counts and rates of contamination of surgical instrument trays.

## Materials and methods

Group assignment

Thirty sterile surgical instrument trays were randomly assigned to either a control or test group (n=15 per group). All trays underwent data collection in a single, unoccupied positive-air-flow OR during normal daytime hours. Data collection was split across two separate weekend days with an equal number of control and test group trays for each day. On each day, the control surgical tray contamination rate was assessed first in the OR under standard conditions. Test surgical tray contamination rate was then assessed after the control group in the OR with the Illuvia™ HUAIRS (HEPA-UV Air Recirculation System) UV-C air filtration device (Aerobiotix, Inc., Miamisburg, OH, USA) running. The Illuvia™ UV-C filtration device was turned on 30 minutes before test group data collection per the manufacturer’s recommendations.

Operating room set-up

Surgical instrument trays were opened using standard sterile technique and placed on top of two sterilely draped back tables. The tables were positioned in the OR in relation to the centrally-placed patient table to simulate a standard total hip arthroplasty. The Illuvia™ UV-C filtration device was positioned diagonally from the air return grates following manufacturer specifications. The position of all equipment in the OR remained the same between groups.

Particle count

A Particles Plus 8303 Handheld Particle Counter (Particles Plus, Inc., Stoughton, MA, USA) was placed atop the OR circulator desk in front of the air return grates. The particle counter was used to obtain the counts of 5µm and 10µm-sized particles within the room; these sizes were selected as they reflect the size range of microbial-carrying particles [18]. Particle counts were obtained immediately after opening surgical trays and every 30 minutes thereafter until an end time point of four hours for each group. Readings were obtained in triplicate at each time point.

Surgical instrument contamination rate

Trays in each group were assessed for contamination using aerobic culture swabs and tubes, as previously described [19]. Each tray was subdivided into four quadrants which were thoroughly swabbed upon opening and every thirty minutes thereafter until an end time point of four hours. All trays remained uncovered during the entire testing period for each group. Cultures were obtained by a single individual who remained sterile in standard OR attire for the entirety of each testing period. To simulate normal OR traffic and airflow disruption, a single individual would pass through the OR from the non-sterile hallway into the sub-sterile core every 10 minutes.

All culture samples were handled by the testing facility’s internal lab. Cultures were kept for a minimum of 72 hours. All species grown from cultures were identified without antibiotic sensitivity data. Trays were considered contaminated upon confirmation of a single positive culture from any quadrant within the tray. The time to contamination and the proportion of trays that remained sterile were recorded for all groups.

Statistical analysis

Statistical analyses were performed using Statistical Package For The Social Sciences (SPSS) software version 22 (IBM Corp., Chicago, IL, USA). Continuous variables were compared using a two-tailed Student’s T-test. Categorical variables were analyzed via Fischer’s exact test. The time to contamination was compared among the groups with the use of a Kaplan-Meier survivorship curve and the log-rank test. Statistical significance was accepted at p<0.05.

## Results

Particle count

Use of the Illuvia™ UV-C filtration unit resulted in significant reductions in the numbers of both the 5µm and 10µm-sized particles when compared with the control. The numbers of 5µm particles were reduced by an average of 64.9% when compared with the control (p<0.001) and the numbers of 10µm particles were reduced by an average of 65.7% (p<0.001) across all time points (Table 1).

**Table 1:** Mean airborne particle counts SD: Standard deviation, UV-C: Ultraviolet C

Particle Size	Control (SD)	UV-C (SD)	P-value
5 µm	3020 (3136)	1059 (1129)	<0.001
10 µm	1373 (1428)	471 (643)	<0.001

Surgical instrument contamination rate

**Figure 1:**
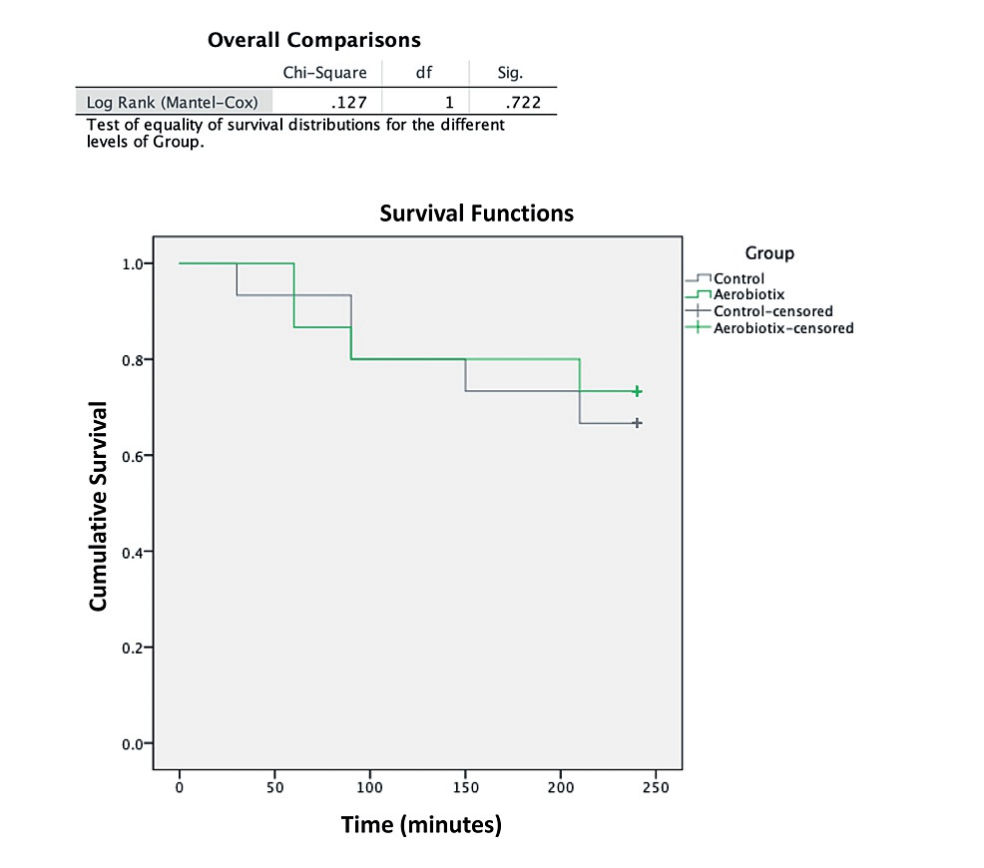
Kaplan-Meier estimates of survival (i.e., absence of bacterial contamination) with log rank (Mantel-Cox) test for surgical instrument trays with the UV-C unit (Aerobiotix) and without (Control). Results of the log rank test are displayed in the above graph.

None of the trays (0/15) yielded positive cultures upon opening at time point zero. The overall contamination rate for the control group was 33.3% (5/15) compared to 26.7% (4/15) for the Illuvia™ UV-C test group (p=1.0). The control group exhibited contamination of one tray at 30 minutes (6.7% total), two trays at 90 minutes (20% total), one tray at 150 minutes (26.7% total), and one tray at 210 minutes (33.3% total). The Illuvia™ UV-C group exhibited contamination of two trays at 60 minutes (13.3% total), one tray at 90 minutes (20% total), and one tray at 210 minutes (26.7% total). The estimated mean survival times for the control and Illuvia™ UV-C groups were 114 and 105 minutes, respectively (Figure 1). When analyzed by the log rank test this was not significantly different (p=0.72).

Only one tray (3.3%) in the control group yielded more than one positive culture at different time points. The nine total trays (including both the control and Illuvia™ UV-C cohorts) that had positive cultures grew: 33% (3/9) *Micrococcus* species, 33% (3/9) *Bacillus* species, 22% (2/9) *Paenibacillus* species, 11% (1/9) *Streptomyces* species, and 11% (1/9) *Staphylococcus epidermidis* (Table 2).

**Table 2:** Organisms grown from contaminated (i.e., bacterial culture positive) surgical instrument trays UV-C: Ultraviolet C

Tray	Time (minutes)	Organism
Control 1	150	Micrococcus luteus
Control 2	30	*Bacillus *species
Control 6	90	Micrococcus luteus
Control 6	240	*Paenibacillus* species
Control 12	210	Paenibacillus urinalis
Control 15	90	Staphylococcus epidermidis
UV-C 7	90	*Micrococcus* species
UV-C 9	60	*Bacillus* species
UV-C 10	60	*Streptomyces* species
UV-C 13	210	*Bacillus* species

## Discussion

The present study sought to determine the effect of a UV-C air filtration device on airborne particulate burden and rates of surgical tray contamination in a simulated OR setting. The results demonstrated that the Illuvia™ UV-C system significantly reduced the number of viable aerosolized particles in the OR, and there was no significant difference in the rates of contamination or “survivability” of opened surgical instrument trays over four hours.

These results suggest that the Illuvia™ UV-C filtration device is successful in reducing air contamination in standard ORs. Devices utilizing HEPA filters have previously been attributed to substantial reductions in air contamination, with some reviews reporting a 99.97% reduction in airborne particles larger than 0.3µm [1,10,17,20,21]. Specifically, in a pilot study assessing the efficacy of the Illuvia™ UV-C filtration unit on particle counts in unoccupied ORs with simulated OR traffic, Curtis et al. demonstrated significant reductions in both viable and total particle counts in ORs containing the device [17]. Similarly, Parvizi et al. reported a 53.4% reduction in OR air contamination following the application of this device [1]. Interventions to reduce air particulate burden in the OR are becoming increasingly important due to the potential effects of air contamination on surgical outcomes [9,10]. However, despite recent engineering standards and practice requirements, the air quality in standard ORs frequently does not reach recommended levels, although there is no universally agreed-upon standard [1,22]. Furthermore, factors such as OR traffic and surgical gowning have been attributed to air contamination that is not adequately controlled by current technologies [13,15,16]. As such, the employment of supplemental systems such as a UV-C air filtration device may be necessary to adequately reduce airborne particulate burdens and improve air quality in modern ORs.

The reduction in air particulates with the Illuvia™ UV-C air filtration system may be partly attributed to the method of air flow as well as the use of UV-C light. Laminar air flow systems have been historically employed in ORs as a method of reducing air contamination and infection rates [6,7,21]. However, recent studies have brought into question the utility of standard laminar air flow filtration devices, noting a potentially increased risk of surgical infections with this type of air delivery [21,23,24]. This increased risk has been attributed to air turbulence that occurs in fringe areas that are not directly underneath the laminar air flow canopies [7,21]. This air can potentially contaminate the tables and instruments that are often located in these areas of the OR. As such, the World Health Organization (WHO) has advised against the use of laminar air flow as a means for reducing surgical infection risk [1]. The Illuvia™ UV-C system employs nonturbulent, unidirectional air flow with a neutral pressure system to filter air in the fringe areas to reduce air entrainment while still avoiding mixing of sterile and contaminated air. In addition, this air filtration system utilizes UV-C germicidal irradiation which has been demonstrated to reduce airborne bacterial counts and inactivate bacterial spores [25,26]. The combination of these mechanisms may have contributed to the substantial decrease in air particulate for the particle sizes known to frequently carry microorganisms.

The reduction of aerosolized particulates in the OR with the Illuvia™ UV-C filtration system may result in reduced surgical site contamination and subsequent risk for postoperative infections. In a study assessing the rate of prosthetic joint infection (PJI) following total knee arthroplasty, Cook et al. demonstrated a substantially decreased rate of PJI in patients who underwent surgery in an OR with an Illuvia™ UV-C system [27]. Darouiche et al. performed a randomized controlled trial assessing airborne contamination and infection rates following total hip arthroplasty, vascular bypass graft implantation, and instrumented spinal procedures performed with and without an air filtration device [10]. The use of the filtration device was associated with a substantial reduction in both airborne colony forming units (CFUs) and postoperative infections, with no reported infections in the air filtration group. Additionally, high density of airborne CFUs was identified as a significant risk factor for infection. In recent years, elevated airborne particulate counts have become more frequently attributed to instrument contamination as well as surgical site and prosthetic infections [9,24,25,27-30]. While the present study was unable to detect a difference in tray contamination between the Illuvia™ UV-C and the control group, this may be attributable to limitations in the number of trays and data collection in an ideal OR setting. However, the substantial reduction in viable airborne particles with the Illuvia™ UV-C unit in the present study may indicate a potential for reducing surgical site contamination and subsequent infections *in vivo*. As such, further clinical trials employing the Illuvia™ UV-C system are necessary to better illustrate the relationship between the device and its effect on surgical infection rates.

This study has several limitations. First, the present study utilized an OR with a positive pressure ventilation system. As such, these results may not be generalizable to all ORs. Second, the cultures and particle counts were obtained in a clean, unoccupied OR that had not been utilized since the previous night. As such, the particle counts and tray contamination rates may be lower than that found in an occupied OR that contains multiple operative cases throughout the day. Third, while there were a large number of cultures obtained, the total number of overall trays and contaminated trays in the present study was low. As such, the power of the study is limited and may have contributed to the inability to detect a difference in contamination rates between the groups. Fourth, contaminated trays as identified by positive cultures cannot be distinguished from potentially false positive results that occur from contamination during culturing in the laboratory. However, the frequency of contaminated trays in the present study is similar to that of a previous study using similar methods [19], suggesting that the contamination rates observed here are likely reliable.

## Conclusions

Postoperative infections are a significant cause of morbidity, mortality, and cost in the current healthcare system. Airborne contamination of surgical wounds and instruments in the OR has been determined to be a significant risk factor for surgical site infections. In order to improve OR air quality, new technologies and policies must be implemented. The present study demonstrated the efficacy of a UV-C air filtration device in reducing microbe-associated airborne particle counts in a simulated OR setting. These results suggest that this device may be effective in mitigating intraoperative airborne contamination, potentially reducing the risk for surgical wound and implant-associated infections. As such, this technology could potentially serve as an adjunctive risk-reduction strategy for patients undergoing orthopedic and other device-related surgical procedures at high risk of environmental contamination. Further clinical trials are necessary to better determine the effect of this air filtration system on postoperative infection rates.
